# 12/15-Lipoxygenase Is Required for the Early Onset of High Fat Diet-Induced Adipose Tissue Inflammation and Insulin Resistance in Mice

**DOI:** 10.1371/journal.pone.0007250

**Published:** 2009-09-29

**Authors:** Dorothy D. Sears, Philip D. Miles, Justin Chapman, Jachelle M. Ofrecio, Felicidad Almazan, Divya Thapar, Yury I. Miller

**Affiliations:** 1 Division of Endocrinology and Metabolism, Department of Medicine, University of California San Diego, La Jolla, California, United States of America; 2 Pfizer Inc., La Jolla, California, United States of America; University of Bremen, Germany

## Abstract

**Background:**

Recent understanding that insulin resistance is an inflammatory condition necessitates searching for genes that regulate inflammation in insulin sensitive tissues. 12/15-lipoxygenase (12/15LO) regulates the expression of proinflammatory cytokines and chemokines and is implicated in the early development of diet-induced atherosclerosis. Thus, we tested the hypothesis that 12/15LO is involved in the onset of high fat diet (HFD)-induced insulin resistance.

**Methodology/Principal Findings:**

Cells over-expressing 12/15LO secreted two potent chemokines, MCP-1 and osteopontin, implicated in the development of insulin resistance. We assessed adipose tissue inflammation and whole body insulin resistance in wild type (WT) and 12/15LO knockout (KO) mice after 2–4 weeks on HFD. In adipose tissue from WT mice, HFD resulted in recruitment of CD11b^+^, F4/80^+^ macrophages and elevated protein levels of the inflammatory markers IL-1β, IL-6, IL-10, IL-12, IFNγ, Cxcl1 and TNFα. Remarkably, adipose tissue from HFD-fed 12/15LO KO mice was not infiltrated by macrophages and did not display any increase in the inflammatory markers compared to adipose tissue from normal chow-fed mice. WT mice developed severe whole body (hepatic and skeletal muscle) insulin resistance after HFD, as measured by hyperinsulinemic euglycemic clamp. In contrast, 12/15LO KO mice exhibited no HFD-induced change in insulin-stimulated glucose disposal rate or hepatic glucose output during clamp studies. Insulin-stimulated Akt phosphorylation in muscle tissue from HFD-fed mice was significantly greater in 12/15LO KO mice than in WT mice.

**Conclusions:**

These results demonstrate that 12/15LO mediates early stages of adipose tissue inflammation and whole body insulin resistance induced by high fat feeding.

## Introduction

Insulin resistance is a pathophysiological condition associated with obesity, aging, and type 2 diabetes that affects skeletal muscle, liver, adipose tissue, and immune cells. Obesity and insulin resistance are associated with macrophage infiltration and inflammation in the adipose tissue of humans and rodent models where a feed-forward cycle of reciprocal adipocyte and macrophage activation results in the secretion of inflammatory proteins and further macrophage recruitment [1], [2]. Pro-inflammatory factors secreted by macrophages and adipocytes are elevated in adipose tissue from obese and type 2 diabetic patients [3]. Adipose tissue inflammation induces insulin resistance through inactivation of insulin receptor substrates (IRS) by cytokine-activated JNK, IKKβ and SOCS [1]. High fat diet (HFD) feeding, a commonly studied model of insulin resistance in rodents, rapidly causes progressive metabolic maladies [4], [5]. Insulin resistance in heart, adipose tissue, liver, and muscle, adipose tissue hypertrophy and inflammatory cell infiltration, and hyperinsulinemia are significantly robust phenotypes observed as early as 1–3 weeks of HFD, with minimal to no total body weight gain [4], [6]–[8]. After 16–20 weeks of HFD, these phenotypes are much more pronounced and additional severe metabolic dysregulations are present including dyslipidemia & ectopic triglyceride storage, hypo-adiponectinemia, adipose tissue hypoxia, cell death and remodeling, beta-cell decompensation, mild hyperglycemia, and deterioration of cardiac function [4]–[6]. The key molecules involved in initiating HFD-induced adipose tissue inflammation and macrophage infiltration are not well characterized. Recent studies suggest an important role for 12/15-lipoxygenase (12/15LO) in monocyte recruitment to and regulation of inflammation in vascular and adipose tissue.

The family of 12/15LO enzymes catalyzes the insertion of molecular oxygen in arachidonic acid (20∶4) at the 12^th^ and/or 15^th^ carbon, resulting in a fatty acid hydroperoxide. 12/15LO oxygenates linoleic acid (18∶2) at the 9^th^ and/or 13^th^ carbon. Free unsaturated fatty acids as well as fatty acids esterified in phospholipids and cholesteryl esters are substrates for 12/15LO. The 12/15LO enzymes are conserved among many plant and animal species and include soybean 15LO, human 15LO (Entrez Gene ID 246), and mouse 12/15LO (Entrez Gene ID 11687). In mammals, 12/15LO is expressed in differentiated macrophages, dendritic cells, inflamed endothelial and smooth muscle cells, and in certain tumors [9]–[14]. There is an emerging understanding that, in both plants and mammals, 12/15LO products are involved in the signaling processes of defense response and inflammation. In plants, 12/15LO initiates the synthesis of jasmonic acid, which regulates defensive genes that respond to wound- and insect-inflicted damage [15], [16]. In mammalian cells, 12/15LO products regulate small GTPases Ras and RhoA, MAP kinases, PKC, and transcription factor NF-κB [12], [17]. Our previous studies suggest that 12/15LO oxidation products activate toll-like receptor-4 (TLR4) in macrophages [18], [19]. Cells that express 12/15LO or are activated with 12/15LO oxidation products produce MCP-1, IL-6, IL-8 and TNFα and induce monocyte binding to endothelial cells [20]–[26].

12/15LO has been implicated in the development of autoimmune diabetes and in vascular complications of diabetes. Arachidonic acid stimulates insulin secretion by β-cells and this process is inhibited by the 12/15LO activity [27]. Moreover, 12/15LO mediates cytokine-induced β-cell damage [28]. Other data suggest that non-obese diabetic (NOD) mice congenic for a targeted deletion of 12/15LO are in fact protected from autoimmune diabetes [29]. A recent study has shown that long-term (8–24 weeks) high fat feeding induces 12/15LO activation and beta cell damage in pancreatic islets, both of which were prevented in 12/15LO KO mice [30]. 12/15LO is also involved in vascular complications of advanced diabetes, such as atherosclerosis and nephropathy, which manifest life-threatening conditions. Specifically under diabetic conditions, vascular smooth muscle cells (VSMC) express 12/15LO which in turn mediates a VSMC switch from a contractile phenotype to a migratory and inflammatory phenotype [9], [22], [31], [32]. This change together with 12/15LO-mediated lipoprotein oxidation and monocyte adhesion to endothelial cells explains the involvement of 12/15LO in the pathogenesis of high fat diet (HFD)-induced atherosclerosis [33]–[36].

Although these previous studies show that 12/15LO plays a role in regulating β-cell survival, advanced diabetic complications and, in a very recent publication, chronic (8–24 weeks) HFD-induced insulin resistance and inflammation [30], the possible involvement of 12/15LO in the early development of insulin resistance has not been studied. Given that 12/15LO is an inflammatory modulator, we asked whether it is required for the onset of HFD-induced insulin resistance. We found that 12/15LO deficiency protected mice from exhibiting elevated inflammatory markers in adipose tissue and, remarkably, prevented whole-body insulin resistance induced by 2–4 weeks of high fat feeding.

## Methods

### Cells

Murine fibroblast cell lines stably over-expressing human 15LO or β-galactosidase (LacZ) [37], [38] were cultured in 10% FBS/DMEM/gentamicin with 0.5 mg/ml G418 (Calbiochem, San Diego, CA) to maintain selection.

### Animals

Male C57BL6 wild type mice were from Jackson Laboratories. Male 12/15LO knockout mice, backcrossed in a C57BL6 background for 10 generations, were a generous gift from Dr. Colin Funk (Queen's University). Starting 16 weeks of age, mice were fed either normal chow (12% kcal from fat; Purina 5001 Lab Diet) of high fat diet (41% kcal from fat; TD96132, Harlan Teklad) for 2 or 4 weeks. All mice involved in clamp studies (wild type and 12/15LO KO, n = 20 each) were singly housed during the two weeks of dietary intervention. This was done in order to ensure equal food access, protect the implanted catheters, and prevent fighting between mice. Mice used for other experiments (acute insulin and adipose tissue FACS studies, n = 20 per strain) were housed 1–3 in a cage. There were infrequent circumstances (∼5 mice total in these studies) when a non-clamp study mouse was singly-housed (for example, an aggressor had to be separated from cage mates to prevent stress and injury to the other mice and to ensure access to food. Mice were housed under controlled light (12∶12 light∶dark) and climate conditions with unlimited access to food and water. All procedures were performed in accordance with the *Guide for Care and Use of Laboratory Animals* of the National Institutes of Health and were approved by the University of California, San Diego, Animal Subjects Committee.

### Analytical methods

Total RNA was isolated from cell lysates using an RNeasy kit from Quiagen (Valencia, CA). Quantitative RT-PCR was performed to measure MCP-1, OPN and GAPDH mRNA levels using a Rotor-Gene RG3000 qPCR machine (Corbett Research, Brisbane, Australia). Primers and probes were from Applied Biosystems (Foster City, CA). The protein levels of MCP-1 in conditioned media were measured by ELISA using a kit from R&D Systems (Minneapolis, MN). The protein levels of OPN in conditioned media were assayed by immunoblot using a primary antibody from Santa Cruz Biotechnology (Santa Cruz, CA).

### In vivo metabolic studies

Insulin sensitivity was assessed in mice fed HFD for 2 weeks using a sub-maximal hyperinsulinemic euglycemic glucose clamp technique as previously described (34), with the following modifications: 1) isoflurane was used for anesthesia during the catheter insertion surgery three days prior to clamp, 2) glucose tracer was infused at 2 μCi/hr during the clamp, and 3) insulin was infused at 3 mU/kg/min during the clamp. The mice were conscious during the clamp and fully recovered after the procedure. Four days later, the mice were fasted for 5 hr, anesthetized (isoflurane) to collect blood by cardiac puncture, and then euthanized (pentobarbital) to collect gastrocnemius muscle, liver, and epididymal adipose tissues. Excised tissues were flash-frozen in liquid nitrogen. Plasma glucose specific activity, glucose disposal rate (GDR), and hepatic glucose output (HGO) were calculated as previously described [39]. In a separate group of mice, acute insulin stimulation was achieved by intraperitoneal injection of 6 hr-fasted mice with 0.85 U/kg insulin. After 15 min, the mice were sacrificed and muscle was harvested as described above.

### Fluorescence-activated cell sorting (FACS) of adipose tissue SVCs

Adipose tissue stromal vascular cells (SVCs) were isolated and analyzed by FACS as previously reported [6] with minor modifications. Briefly, freshly harvested epididymal fat pads were separately rinsed and minced in DPBS +1% BSA and then treated with 1 mg/mL type II collagenase (Sigma, St. Louis, MO) for 25 min in a 37°C shaking water bath. Adipose tissue cell suspensions were filtered through a 100 µm mesh. SVCs were separated from floating adipocytes by centrifugation, incubated in RBC lysis buffer (eBioscience, San Diego, CA) for 5 min, and re-suspended in fresh DPBS +1% BSA. SVCs were incubated with Fc Block (BD Biosciences, San Jose, CA) for 15 min and then stained for 30 min with fluorescent-conjugated antibodies against F4/80 (Ab Serotec, Raleigh, NC) and CD11b (BD Biosciences). Cells were washed two times and re-suspended in DPBS +1% BSA and propidium iodide (Sigma). Presence of the fluorescent stains in the SVCs was analyzed using a FACS Calibur flow cytometer (BD Biosciences). Control SVCs preparations included unstained cells, PI-only stained cells, and fluorescence-minus-one (FMO) stained cells and were used to set gatings and compensation.

### Plasma and tissue analyses

Plasma insulin levels were measured using the Insulin Ultrasensitive (Mouse) EIA method (Alpco, Salem, NH). Muscle lysates were analyzed by western blotting with antibodies against total Akt and phospho-serine Akt (Cell Signaling, Danvers, MA). Signal intensities on chemiluminescence-exposed autoradiographs were densitometrically quantified using a digital Kodak 3D Image station and associated digital image analysis software (Kodak, New Haven, CT). The protein levels of IL-1β, IL-12p70, IFNγ, IL-6, IL-10, Cxcl1 and TNFα in adipose tissue lysates were measured using a multiplex (7-plex) ELISA (Meso Scale Discovery, Gaithersburg, MD).

### Statistical analyses

Student's t-test and ANOVA (and Tukey's post hoc test) were applied for statistical analyses. A p-value cutoff of 0.05 was used to determine statistical significance.

## Results

### 12/15LO expression increases chemokine production

**Figure 1:**
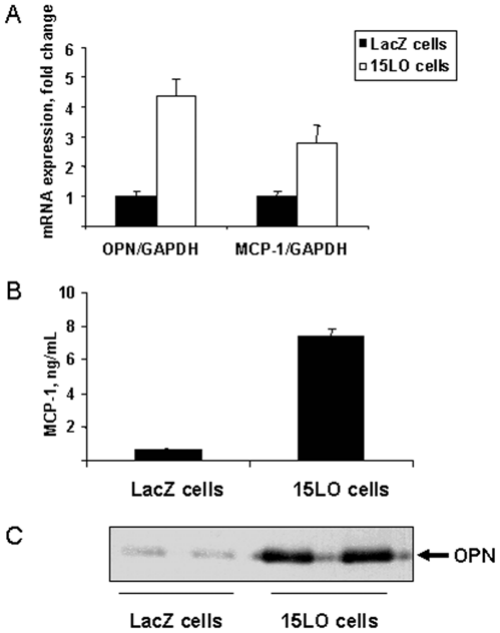
Proinflammatory chemokine production by 15LO-expressing cells. A. Fold difference in OPN and MCP-1 mRNA expression in murine fibroblasts constitutively expressing human 15LO or LacZ. OPN and MCP-1 mRNA levels were measured by qPCR of total RNA and were normalized to GAPDH mRNA. B. Levels of MCP-1 protein in conditioned media from 15LO and LacZ cells, as assayed by ELISA. C. Autoradiograph of OPN protein in conditioned media from 15LO and LacZ cells, as determined by western blotting.

Expression of 12/15LO in various smooth muscle cells and macrophages induces the expression of proinflammatory genes [21], [22], [24], [26]. We used fibroblast cell lines that stably express human 15LO or LacZ (as a negative control) [37] to test whether our 15LO-expressing cells (15LO cells) produce excess pro-inflammatory proteins compared to LacZ-expressing control cells (LacZ cells). We focused on chemokines that could attract monocytes to adipose tissue and have been shown to be involved in the pathogenesis of insulin resistance. Monocyte chemoattractant protein-1 (MCP-1) contributes to macrophage infiltration in adipose tissue and insulin resistance [40], [41]. Osteopontin (OPN) is a proinflammatory cytokine and monocyte chemotactic factor that also mediates obesity-induced insulin resistance [42]. We found that the 15LO cells expressed significantly higher levels of MCP-1 and OPN (mRNA and protein) than the LacZ cells (Figure 1). Expression of other inflammatory mediators TNFα, MIP-2, MIP-1α and IκBζ was not different between the 15LO and LacZ cells (not shown).

### HFD-induced inflammation in adipose tissue is absent in 12/15LO KO mice

**Figure 2:**
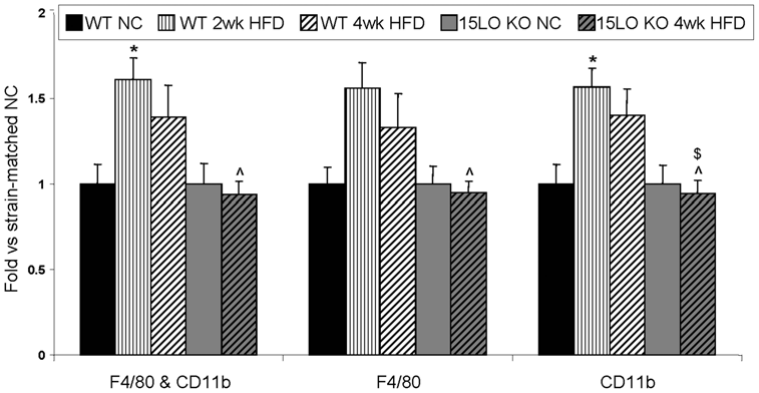
FACS analysis of macrophage content in adipose tissue SVCs. A. HFD-induced fold change in the percent of live adipose tissue SVCs expressing surface markers F4/80, CD11b, and both F4/80 and CD11b. SVCs were isolated from eWAT of mice fed NC, two-week HFD, or four-week HFD. *p<0.05 vs WT NC, ˆp<0.05 vs WT two-week HFD, ^$^p<0.05 vs WT four-week HFD.

Because 12/15LO regulates the expression of monocytic chemokines (Figure 1 and refs. [21], [22], [24]), we compared the effects of short-term high fat feeding on adipose tissue inflammation in C57BL6 wild type (WT) and strain-, gender-, and age-matched 12/15LO knockout (KO) mice. We assessed adipose tissue macrophage infiltration in isolated epididymal white adipose tissue-derived stromal vascular cells (SVCs) using fluorescence-activated cell sorting (FACS), which is a more sensitive and comprehensive method compared to immuno-histochemistry. Because macrophages expressing F4/80 and/or CD11b are increased in adipose tissue after HFD [5], [40], [43], we measured the percentage of live SVCs that express F4/80 or CD11b and the percentage of live SVCs that express both F4/80 and CD11b. The percentage of adipose tissue-derived SVCs expressing F4/80 and/or CD11b was significantly increased in WT mice fed HFD for two weeks (Figure 2) compared to WT mice fed normal chow (NC). This trend of increased macrophage presence also existed after four weeks of HFD. In contrast, adipose tissue-derived SVCs isolated from 12/15LO KO mice fed HFD for four weeks exhibited no change in the percentage of cells expressing F4/80 and CD11b compared with NC-fed 12/15LO KO mice.

**Figure 3:**
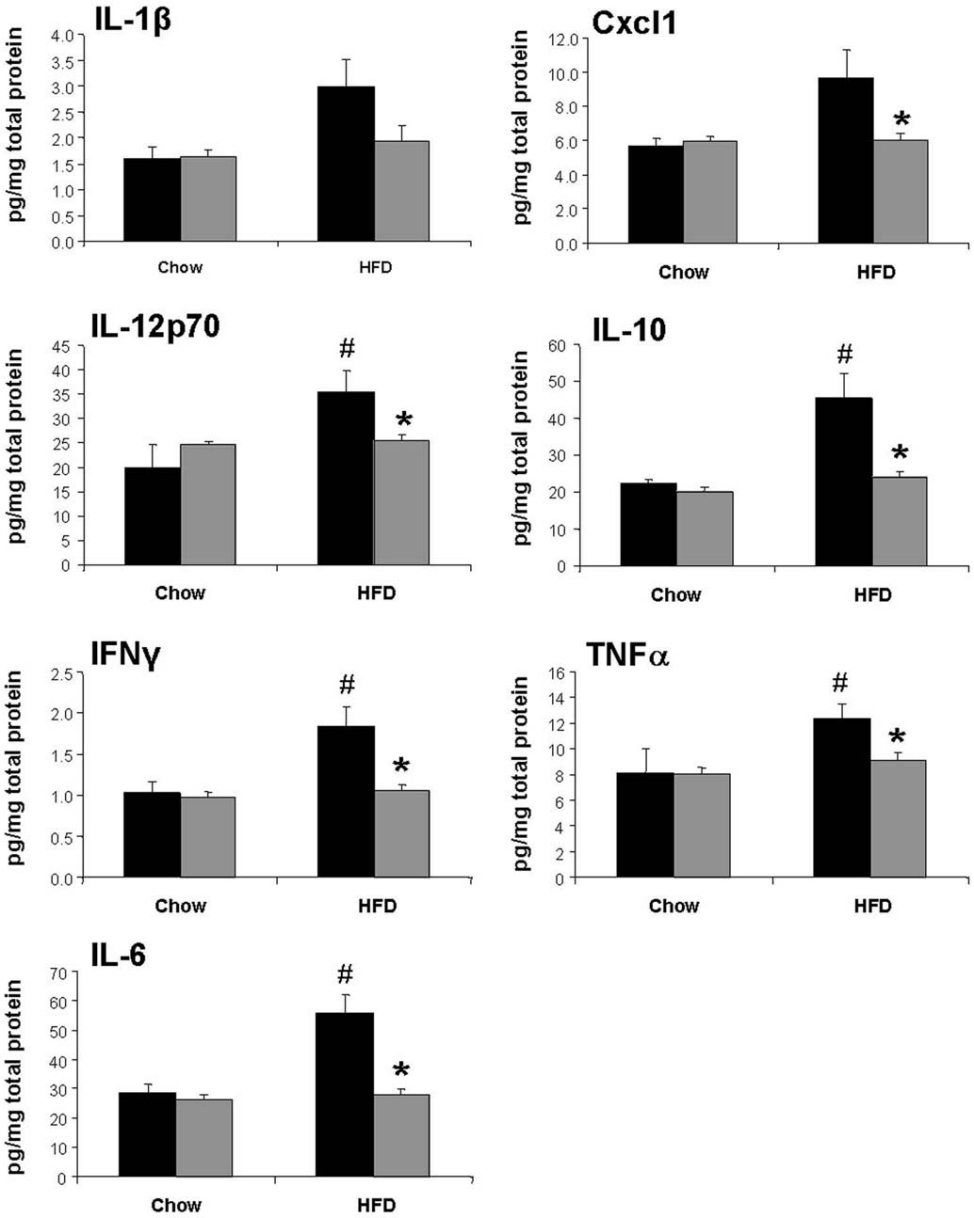
Adipose tissue cytokine levels. Cytokine protein levels measured in eWAT lysates from WT (black bars) and 12/15LO KO (gray bars) mice fed NC or two-week HFD. Values are mean±standard error. 8–10 animals per group. *p<0.05 vs diet-matched WT, ^#^p<0.05 vs strain-matched NC.

We next measured cytokine protein levels in epididymal white adipose tissue (eWAT) lysates from WT and 12/15LO KO mice fed NC or HFD for two weeks. We observed significantly elevated levels of IL-1β, IL-12p70, IFNγ, IL-6, and IL-10 in eWAT lysates from HFD-fed WT mice compared to NC-fed WT mice (Figure 3). Cxcl1 (KC) and TNFα levels also tended to increase after HFD in WT mice but this increase did not reach statistical significance. None of these cytokines were elevated in the plasma of WT mice fed 2-week HFD compared to NC controls. In contrast to WT mice, 12/15LO KO mice were completely protected from HFD-induced increases in IL-1β, IL-12p70, IFNγ, IL-6, IL-10, Cxcl1 and TNFα levels in eWAT. The absence of HFD-induced cytokine elevation in the adipose tissue of 12/15LO KO mice (Figure 3) corresponds with the absence of HFD-induced macrophage infiltration in the adipose tissue of these mice (Figure 2) and supports the notion that, in contrast to WT mice, 12/15LO KO mice fed HFD for two to four weeks do not exhibit macrophage-mediated, adipose tissue inflammation.

### 12/15LO KO mice are protected from HFD-induced insulin resistance

**Figure 4:**
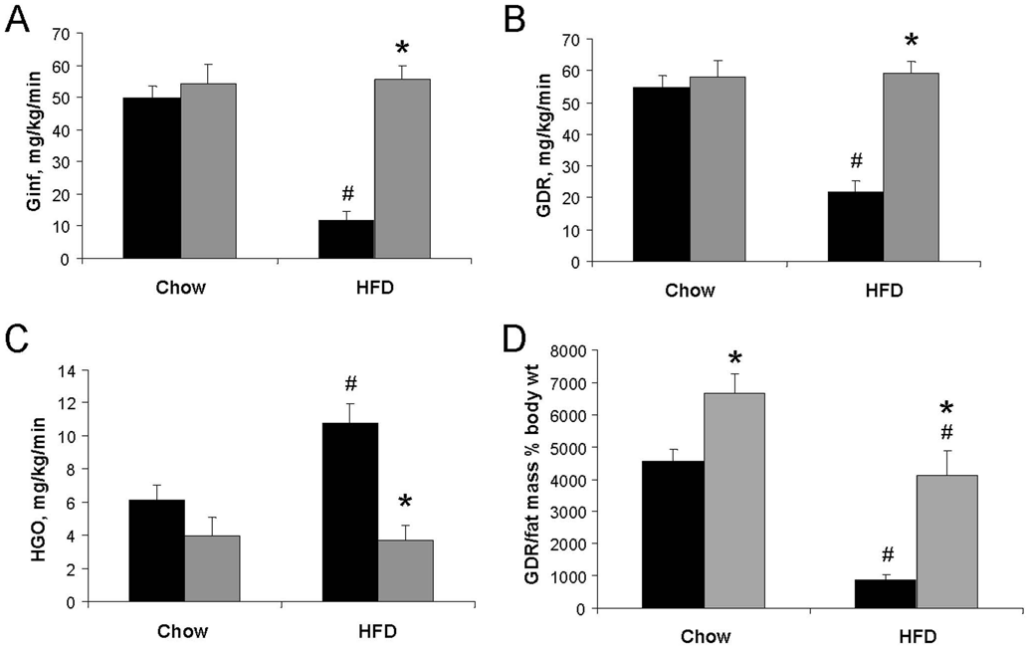
Characterization of whole body insulin sensitivity. Hyperinsulinemic euglycemic clamp studies were conducted to calculate (A) Ginf, (B) GDR, (C) HGO, and (D) GDR normalized to fat pad mass as a percent of body weight in WT (black bars) and 12/15LO KO (gray bars) mice fed NC or two-week HFD. Values are mean±standard error. 7–9 animals per group. *p<0.05 vs WT HFD, ^#^p<0.05 vs WT NC.

We investigated whether the absence of HFD-induced adipose tissue inflammation in 12/15LO KO mice correlated with protection from whole body insulin resistance. We conducted euglycemic hyperinsulinemic clamp studies on WT and 12/15LO KO mice fed NC or HFD for two weeks. High fat feeding induced significant differences in clamp data from HFD-fed compared to NC-fed WT mice, specifically, 76% lower glucose infusion rate (Ginf), 60% lower glucose disposal rate (GDR), and 76% higher hepatic glucose output rate (HGO) (Figure 4A–C). 12/15LO KO mice were completely protected from the severe HFD-induced changes in Ginf, GDR, and HGO that we observed in WT mice. There was no significant difference in basal glucose turnover rate (basal HGO = basal GDR) between the NC-fed WT (15.9±2.2 mg/kg/min) and 12/15LO KO mice (16.0±1.9 mg/kg/min) or between the WT NC-fed and WT HFD-fed mice (15.7±1.9 mg/kg/min). The clamp study data indicate that 12/15LO deficiency provides protection from HFD-induced hepatic and skeletal muscle insulin resistance. HFD-fed 12/15LO KO mice did exhibit a similar fold increase in eWAT mass but not hyperinsulinemia, compared to HFD-fed WT mice (Table 1). To demonstrate that there was no direct dependency between adiposity and insulin resistance in WT and 12/15LO KO mice, we normalized the GDR values to the fat pad mass as a % of body weight. Figure 4D shows that, even after this normalization, there remains a dramatically different insulin sensitivity between WT and 12/15LO KO mice fed chow or high-fat diet.

**Table 1:** Mouse strain characteristics.

	WT			12/15-LO KO		
	NC	HFD	Fold Change	NC	HFD	Fold Change
**Whole body weight, g**	26.4 (0.5)	31.2 (0.7) **#**		25.2 (0.9)	25.2 (0.8) *	
**Epididymal fat pad weight, g**	0.32 (0.02)	0.78 (0.06) **#**	2.45 (0.20)	0.23 (0.01)	0.43 (0.06) #, *	1.89 (0.26), **ns vs WT HFD**
**Epididymal fat pad mass, % body weight**	1.20 (0.08)	2.49 (0.19) #	2.07 (0.16)	0.89 (0.04)	1.64 (0.18) #, *	1.84 (0.20), **ns vs WT HFD**
**Fasting plasma glucose, mg/dL**	160 (5)	174 (6)		177 (5)	202 (9) #, *	
**Fasting plasma insulin, ng/mL**	0.51 (0.18)	2.28 (0.83) #		0.63 (0.22)	0.57 (0.18) *	

Data are averages±standard error. 7–10 mice per group. * p<0.05 vs diet-matched WT, # p<0.05 vs strain-matched NC. ns - not significant.

### Diminished insulin-activated Akt phosphorylation in muscle from HFD-fed WT compared to 12/15LO KO mice

**Figure 5:**
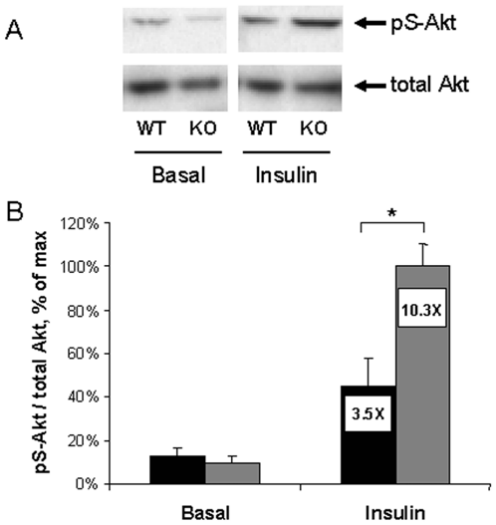
Basal and insulin-stimulated Akt phosphorylation in muscle of HFD-fed mice. WT (black bars) and 12/15LO KO (gray bars) mice were fed HFD for two weeks. Mice were then injected intraperitoneally with 0.85 U/kg insulin or vehicle. After 15 min, mice were sacrificed and gastrocnemius muscles were rapidly isolated. Phospho-Ser^473^ Akt and total Akt levels in muscle lysates were measured by western blotting. A. Representative western blot images. B. Quantitation of all Akt western blot images. Phospho-Ser^473^ Akt/total Akt ratio values were normalized to the maximal signal observed. Bar graph values are mean±standard error. Insulin-induced fold changes in Akt phosphorylation are shown within the bars. 2–6 animals per group. *p<0.05.

In order to assess the effects of 12/15LO KO on insulin signal transduction in skeletal muscle from mice fed HFD, we examined acute insulin-activated Akt phosphorylation in a separate group of mice. Serine-phosphorylation of Akt in muscle lysates (phospho-Ser^473^ Akt normalized to total Akt protein) was determined by western blot using primary antibodies for total Akt protein and phospho-Ser^473^ Akt. As expected, acute insulin treatment induced phosphorylation of Akt in both groups (Figure 5). However, the absolute level of insulin-stimulated Akt phosphorylation and the insulin-stimulated fold change in Akt phosphorylation were both significantly greater, two- and three-fold, respectively, in 12/15LO KO mice compared to WT mice. Because activation (phosphorylation) of Akt is a critical early event in the insulin receptor signaling, these data corroborate our *in vivo* results (Figure 4B) demonstrating greater insulin sensitivity in the muscle of HFD-fed 12/15LO KO mice compared to WT mice.

## Discussion

HFD-induced and obesity-related insulin resistance is associated with chronic low grade inflammation in adipose tissue characterized by macrophage infiltration and elevation of inflammatory cytokine expression. HFD-induced effects on adipose tissue inflammation are initiated early in the course of high fat feeding and are coincident with insulin resistance [5], [6]. Although the known biological roles of 12/15LO include regulating inflammation, a role for 12/15LO in the early development of HFD-induced adipose tissue inflammation and/or whole body insulin resistance has not been described. 12/15LO is expressed in all vascular cell types, including endothelial cells, macrophages and VSMCs, and its expression is elevated under inflammatory conditions. Therefore, we hypothesized that 12/15LO might regulate HFD-induced adipose tissue inflammation. Indeed, we found that high fat feeding induced macrophage infiltration into adipose tissue of WT but not 12/15LO KO mice, presumably, via 12/15LO-regulated chemokine secretion. The difference in adipose tissue macrophage infiltration between HFD-fed WT and 12/15LO KO mice corresponded with their difference in adipose tissue inflammatory cytokine elevation. In addition to being protected from adipose tissue inflammation, HFD-fed 12/15LO KO mice were also dramatically protected from hepatic and skeletal muscle insulin resistance compared to HFD-fed WT mice.

Adipose tissue macrophages are derived from circulating monocytes that attach to and migrate through endothelial cells (ECs) in the tissue microvasculature. 12/15LO expression and activity is increased in atherogenic and hyperglycemic diabetes models [12], [36], [44], [45], conditions similar to the postprandial state of non-diabetic, diet-induced insulin resistance in which case hyperlipidemia and hyperglycemia are more pronounced and sustained. In these model conditions, activated 12/15LO regulates monocyte attachment to ECs in part by increasing expression of adhesion molecule ICAM-1 on the surface of ECs via activation of RhoA and NF-κB [12], [36]. 12/15LO increases the expression of chemokines MCP-1 and OPN (our data presented here and [21], [24], [26]). Thus, 12/15LO-mediated regulation of chemoattractants and an adhesion receptor may account for the increased monocyte infiltration into adipose tissue that we observed in HFD-fed WT but not 12/15LO KO mice.

Both WT and 12/15LO KO mice exhibited an approximate two-fold increase in eWAT mass during the two-week high fat feeding period. Although HFD-induced eWAT expansion in 12/15LO KO mice was somewhat blunted compared to WT mice, the fold change in mass compared to NC-fed controls was statistically indistinguishable from that in WT mice. Despite similar eWAT expansion, the number of macrophages and levels of pro-inflammatory cytokines in eWAT from 12/15LO KO mice were completely unchanged by HFD, and significantly different from the elevated levels we observed in HFD-fed WT mice. Our findings suggest that the proinflammatory effects of 12/15LO expression observed by Nunemaker, et al. after 8 and 24 weeks of HFD [30] in fact occur significantly earlier during the initial stages of HFD-induced insulin resistance (2–4 weeks). Chakrabarti, et al. have recently demonstrated that 12/15LO is up-regulated in adipose tissue from high fat fed mice and that 12/15LO products induce inflammation and insulin resistance in 3T3-L1 adipoctyes [46]. HFD-induced elevation of eWAT cytokine levels could originate from macrophages, adipocytes, endothelial cells and/or preadipocytes within adipose tissue. We speculate that pro-inflammatory macrophages and adipocytes are the most likely sources of elevated eWAT cytokines in high fat diet-fed WT mice.

We used a two- and four-week high fat feeding protocol because it significantly induces the phenotypes of insulin resistance and adipose tissue hypertrophy and inflammation in WT mice without causing the severe metabolic dysfunctions that manifest after longer high fat feeding [4]–[8]. Mouse phenotypes induced by our high fat feeding protocol were similar to those observed in other time course studies of HFD-induced adipose tissue inflammation and insulin resistance [4]–[6]. Two-week high fat feeding induced hyperinsulinemia and severe hepatic and skeletal muscle insulin resistance in WT but not 12/15LO KO mice. 12/15LO KO mice exhibited significantly greater insulin-stimulated skeletal muscle Akt phosphorylation after HFD, compared to WT mice, corresponding to their greater insulin sensitivity. Given the cross-talk between adipose tissue, liver, and skeletal muscle that affects insulin sensitivity [1], [47], protection from adipose tissue inflammation in the 12/15LO KO mice may be the primary site of action leading to protection from whole body insulin resistance.

In summary, we find that 12/15LO is a key modulator of the onset of high fat diet-induced insulin resistance in liver, muscle and adipose tissue. We provide evidence that the mechanism by which the 12/15LO KO mice are protected from the initial stages of HFD-induced insulin resistance involves suppression of adipose tissue pro-inflammatory macrophage infiltration and inflammatory cytokine elevation. 12/15LO is a key participant in the development of diet-induced insulin resistance and, thus, a viable therapeutic target for the treatment of human insulin resistance and type 2 diabetes.
